# Efficacy and underlying mechanisms of *three-circle post standing qigong* on insomnia in college students: a four-arm, double-blind, randomized controlled trial protocol

**DOI:** 10.1186/s12906-024-04544-9

**Published:** 2024-06-18

**Authors:** Ming-min Xu, Nan Li, Tian-nuo Hu, Zheng-kun Zhou, Yue Chen, Xue Huang, Yulong Wei, Yu Guo

**Affiliations:** 1 School of Traditional Chinese Medicine, Jinan University, Guangzhou, China; 2 International Liaison Department, World Federation of Chinese Medicine Societies, Beijing, China; 3 School of Acupuncture-Moxibustion and Tuina, Beijing University of Chinese Medicine, Beijing, China; 4 Yangfang Outpatient Department, Northern District of People’s Liberation Army General Hospital, Beijing, China; 5 Guangzhou Key Laboratory of Formula-Pattern of Traditional Chinese Medicine, School of Traditional Chinese Medicine, Jinan University, Guangzhou, China

**Keywords:** Insomnia, College students, Three-circle Post Standing Qigong, Randomized clinical trial, Therapeutic clinical effects and underlying mechanisms

## Abstract

**Background:**

Insomnia is common in college students, but its impact on health and wellbeing is often neglected. Enhancing sleep quality through targeted interventions could improve overall health and reduce the risk of consequent co-morbidities and mental health problems. Qigong exercises have been shown to significantly improve sleep quality and relieve insomnia. *Three-circle Post Standing* (TCPS) can help integrate body, breath, and mind, a fundamental principle of Qigong that promotes holistic wellbeing. In this clinical trial, we aim to (1) evaluate the feasibility, safety, and therapeutic efficacy of administering TCPS to improve sleep quality and quality of life in college students with insomnia; (2) explore the neurophysiological mechanisms underlying the mind adjustments mediated by TCPS in insomnia; (3) investigate body and breath pathophysiology mediated by TCPS in insomnia; and (4) assess the long-term efficacy of TCPS in terms of sleep quality and quality of life.

**Methods:**

This will be a prospective, parallel, four-arm, double-blind randomized controlled trial to investigate the effects and underlying mechanisms of TCPS on college students with insomnia. One hundred college students meeting diagnostic criteria for insomnia will be randomly assigned to receive either 14 weeks of standardized TCPS training (two weeks of centralized training followed by 12 weeks of supervised training) or sham-control *Post Standing* training. Efficacy outcomes including sleep quality, quality of life, neurophysiological assessments, plantar pressure, biomechanical balance, and physical measures will be collected at baseline, eight weeks (mid-point of supervised training), and 14 weeks (end of supervised training). Sleep quality and quality of life will also be evaluated during the four- and eight-week follow-up.

**Discussion:**

This trial will be an important milestone in the development of new therapeutic approaches for insomnia and should be easily implementable by college students with insomnia. The neuro- and pathophysiological assessments will provide new insights into the mechanisms underlying TCPS.

**Clinical trial registration:**

This trial has been registered in the China Clinical Trials Registry (registration number: ChiCTR2400080763).

## Introduction

College years are often a period of transition for students, marked by significant physical, psychological, and interpersonal changes and altered living and learning environments. Insomnia is common in many students during this period, with about a third of college students reporting getting less than seven hours of sleep per night and nearly two thirds describing poor sleep quality [1]. Similarly, in China, insomnia has been reported in 17% of college students [2]. College students with insomnia often complain that reduced sleep time and/or quality of sleep seriously affect their quality of life, daytime functioning, and academic performance, especially through fatigue, dizziness, headache, malaise, frequent mood lability, school absenteeism, and even accidents and injuries [3–5]. Furthermore, chronic insomnia can increase the risk of developing health problems such as obesity [6], diabetes [7], high blood pressure [7], coronary heart disease [8], and psychological disorders [3, 5] and exacerbate existing medical conditions and contribute to the development of suicidal thoughts [3, 9].

Most current clinical guidelines recommend short-term hypnotic medications for insomnia (up to 4 weeks) due to the risk of tolerance and dependence. These medications can cause notable side-effects such as hangover, morning grogginess, unusual sleep behaviors, memory/cognitive impairments, arrhythmia, fatigue, and rebound insomnia upon cessation [10, 11]. Cognitive behavioral therapy for insomnia (CBT-I) is recommended globally as first-line treatment for insomnia [10–12], but the high cost and a lack of trained therapists restrict access of CBT-I to many college students. In addition, adherence to and efficacy of CBT-I can be limited. A safe and effective mind–body intervention for insomnia is urgently needed for this population.

Qigong exercises are an important part of traditional Chinese medicine (TCM) that have attracted scientific and medical attention due to their potential mind–body health benefits [13, 14]. Recent high-quality randomized controlled trials (RCTs) and meta-analyses have demonstrated efficacy for Qigong exercises including *Tai Chi* [15], *Baduanjin* [16, 17], *Guolin Qigong* [18], and *Chan-chuang Qigong* [19] for effectively treating insomnia and significantly enhancing sleep quality. However, these Qigong exercises are all dynamic exercises incorporating the three fundamental “adjustments” of TCM Qigong theory—body, breath, and mind—where the goal is integrating these three elements and ultimately attaining a state of harmonious unity and holistic wellbeing [13, 14]. Beginners, especially those with poor coordination, may focus more on their movements rather than their breathing and mental states, and it often takes them longer to master dynamic exercises compared to static exercises in Qigong.

*Post Standing*, a static Qigong exercise, also known as "Zhan Zhuang", "standing stake", or "standing meditation", originated from the ancient Chinese proverb "stand alone and guard your spirit" found in "Plain Questions—On Health-Keeping of Remote Antiquity" or Su Wen—Shang Gu Tian Zhen Lun (素问·上古天真论). This ancient exercise is considered a fundamental posture and practice in various Qigong exercises. Over its long history, *Post Standing* has branched into various styles, where *Three-circle Post Standing* (TCPS) is a highly regarded and frequently practiced form of *Post Standing*. The salient characteristic of TCPS is the establishment of three distinct and interconnected circular configurations via the *Post Standing* stance, including the hand circle, which mimics the act of holding an open-fingered ball, the arm circle, which resembles grasping a tree, and the foot circle, which involves rotating the toes inward to create a semicircle [20]. During these exercises, abdominal breaths should be deep and regular [21], and the subject should concentrate on the three circular configurations with a relaxed demeanor [22]. TCPS can help practitioners achieve physical alignment and biomechanical balance that is more conducive to optimizing their exercises and integrating body, breath, and mind for harmonious unity.

Compared to pharmaceutical and psychological interventions, TCPS prioritizes holistic psychological wellness and emotional stability. It offers a proactive and self-practiced approach that allows individuals to regulate their own mind–body connection, unlocking their inner potential without constraints of time or space [22]. This makes TCPS a convenient and safe exercise for enhancing emotional stability, improving sleep health, and preventing psychosomatic disorders. However, there are currently few high-quality clinical studies of the effects of TCPS on insomnia. We have therefore designed a rigorous sham-controlled double-blind clinical trial with the primary objective of evaluating the efficacy of TCPS for treating insomnia in college students. Secondary objectives included assessing plantar pressure and biomechanical equilibrium, respiratory patterns, and resting-state brain activity in individuals engaging in TCPS. In conducting this comprehensive neurophysiological and pathophysiological analysis, we aim to evaluate whether individuals meet standards of TCPS and investigate the underlying mechanisms by which TCPS may impact insomnia.

This study aims to: (1) assess the feasibility, safety, and clinical efficacy of TCPS on sleep quality and quality of life in college students with insomnia in the sham-*Post Standing* controlled double-blind setting; (2) explore the neurophysiological mechanisms underlying the mind adjustments occurring in TCPS associated with insomnia by investigating the functional connectivity between brain regions through resting-state electroencephalography (EEG); (3) investigate the impact of body and breath adjustments in TCPS on the physical function of college students with insomnia by evaluating respiratory function, heart rate variability (HRV), blood pressure, and oxygen saturation; and (4) assess the long-term efficacy of TCPS in terms of sleep quality and quality of life.

Our primary hypothesis is that TCPS will improve sleep quality and quality of life of college students with insomnia compared with students engaging in sham-controlled *Post Standing* practice and that it will be safe, acceptable, and feasible. Our secondary hypotheses are that (i) the mind adjustment of TCPS will increase alpha and theta frequencies in specific brain regions, particularly in the alpha band, and enhance functional connectivity prior to observable improvements in sleep quality; and (ii) engaging in TCPS will modulate HRV, respiratory function, blood pressure, and oxygen saturation due to body and breath adjustments. Ultimately, we also expect TCPS to improve sleep quality and overall quality of life of students with insomnia following the intervention.

## Methods/design

This protocol was designed according to the Standard Protocol Items: Recommendations for Interventional Trials (SPIRIT) guidelines [23].

### Trial design

This will be a prospective, double-blinded, randomized controlled trial with a parallel four-arm design. Participants will be randomized to either TCPS or one of three sham-controlled *Post Standing* exercises. Participants and data collection/analysis personnel will be blinded to the intervention allocation.

### Study setting and procedure

All participants will be college students with insomnia admitted to the main campus (Shipai campus) of Jinan University, Guangzhou City, China. One hundred participants meeting the trial criteria will be included and randomly assigned to the standard TCPS group or three sham-controlled *Post Standing* groups according to a 1:1:1:1 allocation ratio with allocation concealment. The training regimen will include both centralized (two weeks) and supervised (12 weeks) training and a follow-up period of eight weeks, i.e., a total study duration of 22 weeks.

The primary outcomes will be measured using EEG data [24–27] and Pittsburgh Sleep Quality Index (PSQI) scores [28, 29] to evaluate central nervous system (CNS) bioelectrical activity and sleep quality, respectively. The secondary outcomes will include Insomnia Severity Index (ISI) scores [30, 31], 36-Item Short Form Health Survey Questionnaire (SF-36) scores [32, 33], plantar pressure and biomechanical equilibrium [34, 35], and physical function including respiratory function [36, 37], HRV [38, 39], blood pressure [40, 41], and blood oxygen saturation [42, 43].

**Fig. 1:**
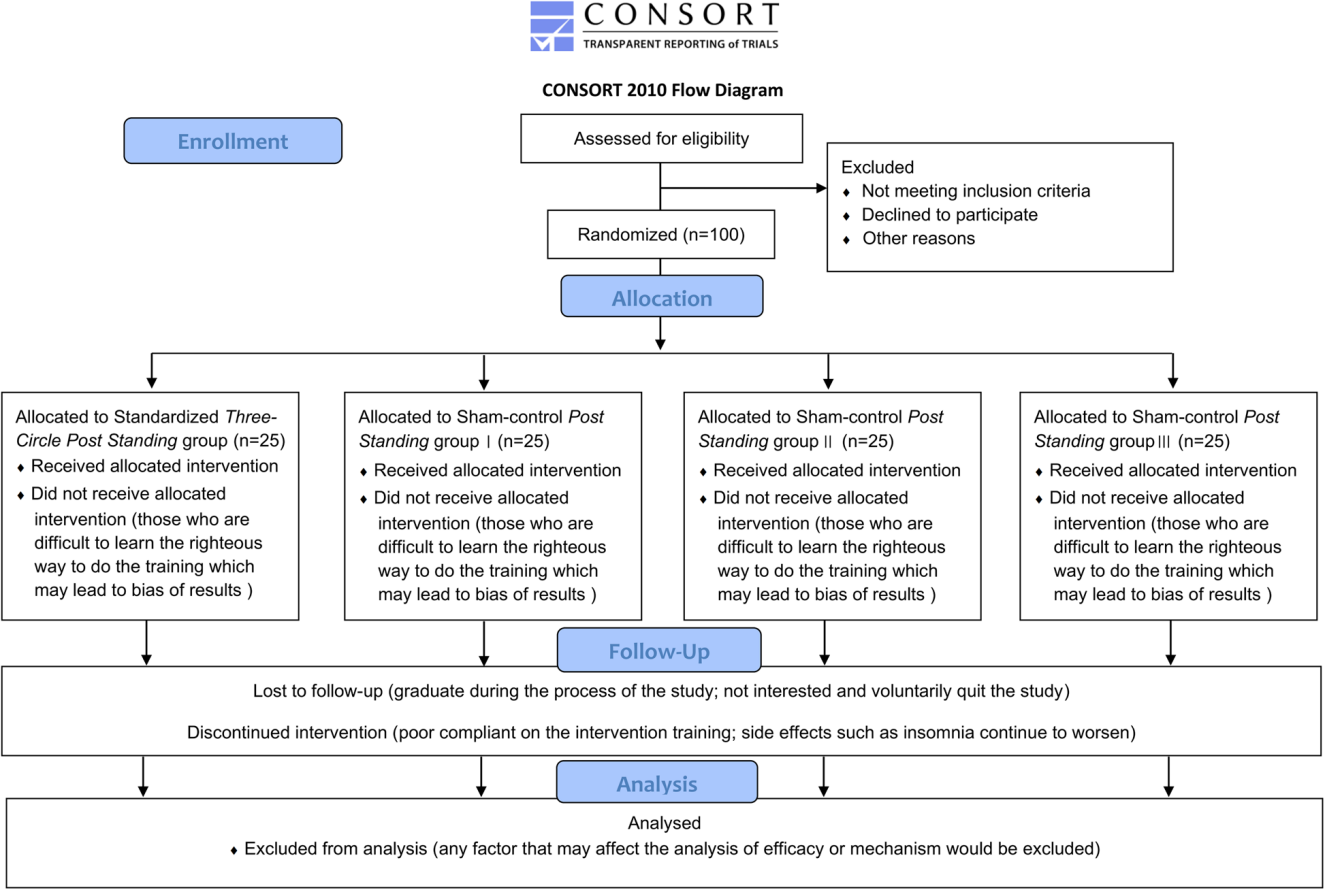
Flow diagram study design

All outcomes will be measured at baseline, at eight weeks (at the mid-point of supervised training), and at 14 weeks (at the end of supervised training). Sleep quality and quality of life will also be evaluated during the four- and eight-week follow-up periods. All measurements will be conducted in a professional exercise and human science laboratory. The study design is shown in Fig. 1 and the timetable is shown in Table 1.

**Table 1:** Schedule of enrolment, assessments, and intervention training

Study phase	Baseline period		Intervention training period					Follow-up period	
	**Enrollment**	**Allocation**	**Centralized training**	**Supervised training**	**Outcomes assessment**	**Supervised training**	**Outcomes assessment**		
**Timepoint (week)**	**-1**	**0**	**1–2**	**3–8**	**8**	**9–14**	**14**	**18**	**22**
**Enrollment and baseline survey data collection**									
Eligibility Criteria screening	**√**								
Informed consent	**√**								
General demographic characteristics	**√**								
Sleep quality evaluation	**√**								
Neurophysiological assessments	**√**								
Plantar pressure, biomechanical balance, and physical function assessments	**√**								
Randomization		**√**							
Allocation		**√**							
**Interventions training**									
Standardized TCPS group (TCPS posture with abdominal breath adjustment)			**√**	**√**		**√**			
Sham-control *Post Standing* group I (TCPS posture with natural chest breath adjustment)			**√**	**√**		**√**			
Sham-control *Post Standing* group II (normal standing posture with abdominal breath adjustment)			**√**	**√**		**√**			
Sham-control *Post Standing* group III (normal standing posture with natural chest breath adjustment)			**√**	**√**		**√**			
**Feasibility and compliance evaluation**									
Training self-evaluation log			**√**	**√**		**√**			
Training sign-in sheet			**√**	**√**		**√**			
Physical activity, sedentary time, and sleeping time			**√**	**√**		**√**		**√**	**√**
**Safety evaluation**									
Adverse events			**√**	**√**	**√**	**√**	**√**		
Serious adverse events			**√**	**√**	**√**	**√**	**√**		
**Efficacy outcome evaluation**									
Sleep quality (PSQI, ISI)		**√**			**√**		**√**	**√**	**√**
Quality of life (SF-36)		**√**			**√**		**√**	**√**	**√**
**Mechanism outcomes assessment**									
The bioelectrical activity generated by the CNS		**√**			**√**		**√**		
Plantar pressure and biomechanical balance		**√**			**√**		**√**		
Respiratory function		**√**			**√**		**√**		
HRV		**√**			**√**		**√**		
Blood pressure		**√**			**√**		**√**		
Blood oxygen saturation of blood		**√**			**√**		√		

*Abbreviations: CNS* central nervous system, *TCPS Three-circle Post Standing*, *PSQI* Pittsburgh Sleep Quality Index, *ISI* Insomnia Severity Index, *SF-36, 36*-Item Short Form Health Survey Questionnaire, *HRV* heart rate variability

### Sample size estimation

Sample size calculations were performed a priori using G-Power Software (v3.1.9.7, Germany). Due to limited previous studies on the use of *Post Standing* or other Qigong exercises in participants with insomnia, the effect size calculation was based on the best available data regarding the hypothesized change in PSQI score found in the literature. The PSQI effect size ranged from 0.31 to 0.83, so we adopted a conservative estimate of 0.31 for our calculation [44]. An F-test ANOVA (repeated measures, within-between interaction) was used for four groups and three measurements. The desired power was set at 80%, with a two-sided α error probability of 0.05. This resulted in a required sample size of 80 participants. Considering a potential attrition rate of 20%, we aim to recruit a minimum of 100 participants for this study. To allocate an equal number of participants to each arm (1:1:1:1), a minimum of 25 participants per arm is necessary.

### Eligibility criteria

This study aims to recruit full-time college students aged 18 to 26 with normal hearing and right-handedness who meet the Diagnostic and Statistical Manual of Mental Disorders, Fifth Edition (DSM-V) criteria for insomnia disorder. They must have sleep difficulties at least three times per week for a minimum of three months, with impaired daytime functioning and a PSQI score above 5 and an ISI score above 7. Participants must be willing to participate, understand study requirements, and provide informed consent in accordance with the Declaration of Helsinki. The inclusion and exclusion criteria are shown in Table 2.

**Table 2:** Inclusion and exclusion criteria

Inclusion criteria	Exclusion criteria
**Eligible participants who meet the following criteria will be included: Participants matching any of the following criteria will be excluded:**	
• Full-time college students, aged between 18 and 26 years • Right-handed, either sex • Normal or corrected to normal hearing	• Engaging in long-term regular practice (regular practice defined as a frequency of at least three times a week and at least 30 min per session) of *Post Standing* or other traditional Chinese mind–body exercises, or any kind of physical activity and muscle strength training at least six months prior to study enrollment
• Fulfilling the diagnostic criteria for insomnia disorder (including difficulty in initiating sleep, maintaining sleep, or early morning awakening with inability to return to sleep, complaints of impaired daytime functioning and sleep difficulty occurring at least three times per week for a minimum of 3 months) according to the DSM-V	• Being in a member of martial arts, dance, aerobics, sanda, taekwondo, and other similar clubs or associations
• PSQI score > 5 and ISI score > 7 • Willing to participate in the study and understand and provide signed informed consent forms according to the Declaration of Helsinki	• Presence of secondary insomnia caused by smoking addiction, drug or alcoholic abuse or dependence, stress disorder, personality disorder, psychoactive substances and non-addictive substances, neurological or psychiatric disorders or other underlying diseases or symptoms such as cervical spondylosis, fever, cough, and pain symptoms
• Able to understand the study process, complete weekly sessions/modules of the intervention program on a regular basis, cooperate with the scale assessments and measurement of EEG, plantar pressure and biomechanical balance, and physical function	• Presence of another sleep–wake disorder (e.g., narcolepsy, restless leg syndrome, a breathing-related sleep disorder, a circadian sleep–wake rhythm disorder, a parasomnia) • Irregular learning and living schedules over the prior four weeks such as staying up late or internet addiction • Have a history of chronic headache and migraine, primary and secondary brain injury, head trauma, and familial psychosis • Taking any medication or healthcare products or receiving other sleep-related non-pharmacotherapy to improve sleep quality at least two weeks prior to study entry • Suffering from malignant tumors or other serious consumptive diseases, autoimmune diseases, infectious diseases, neurological or psychiatric disorders, serious organic disease, or other chronic physical diseases including cardiovascular disease, pulmonary, renal, hepatic or gastrointestinal disease, or hematopoietic system diseases, musculoskeletal system diseases, or other contraindications to exercise • Pregnant, breastfeeding, or planning to conceive within the study period
	• Implantation of active metal or pacemaker or defibrillator in the body that affects measurement and assessment • Skin integrity at the electrode placement site is impaired or allergic to electrode gel or adhesive • Unable to perform breath training due to the onset of acute respiratory disease or other reasons • Participated in any other academic studies or clinical trials in the past 8 weeks or participating in any other academic studies or clinical trials during the study period • Any other circumstances that the investigators believe may make the participant unable to complete, comply with, or unsuitable for this study

*Abbreviations: DSM-V* Diagnostic and Statistical Manual of Mental Disorders, Fifth Edition, *PSQI* Pittsburgh Sleep Quality Index, *ISI* Insomnia Severity Index, *EEG* electroencephalogram

## Intervention training methods

### Standardized TCPS group (TCPS posture with abdominal breath adjustment)

**Fig. 2:**
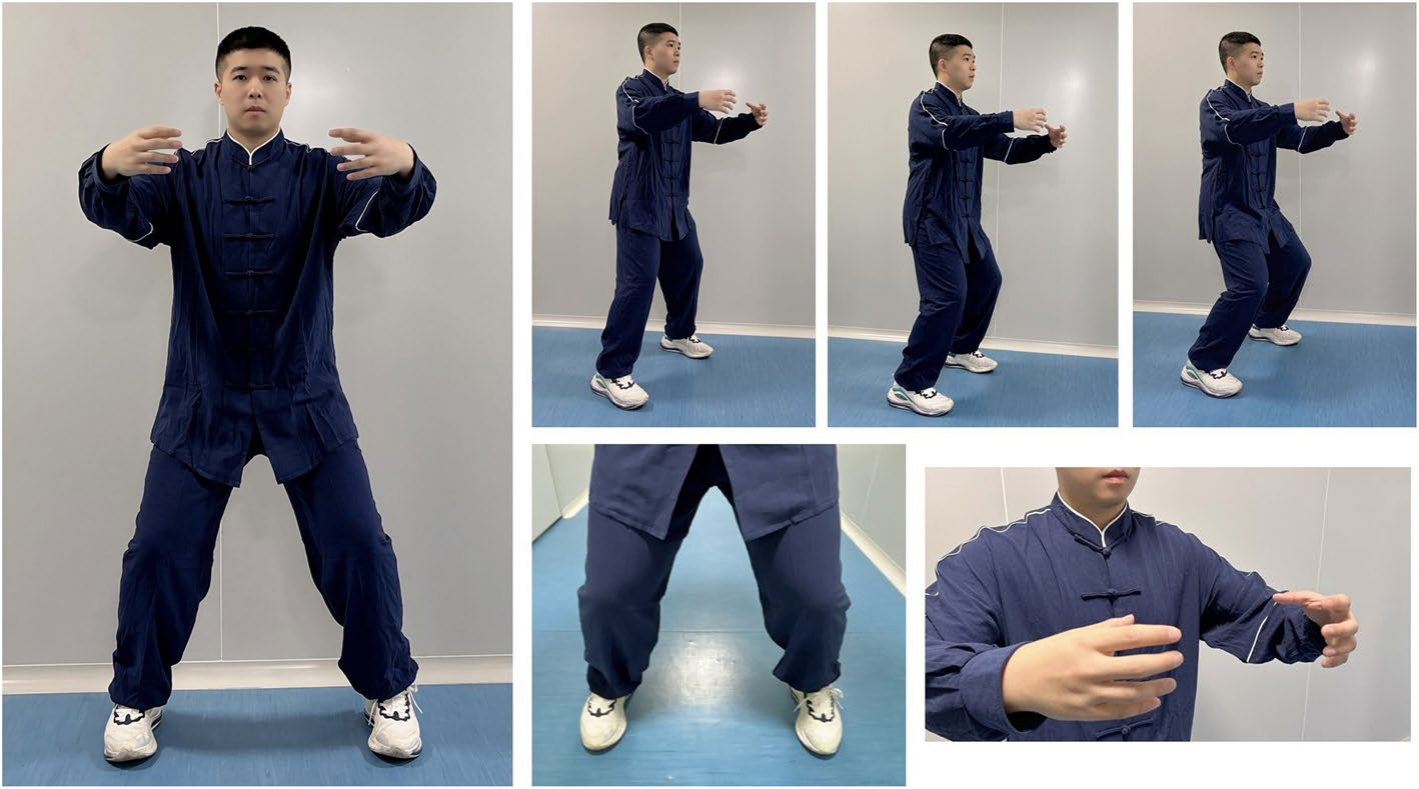
The posture and body adjustments of standardized TCPS

Standardized TCPS group training will consist of three elements: body, breath, and mind adjustments. As seen in the standardized *TCPS* shown in Fig. 2, body adjustment consists of ball encircling training, which requires holding the arms as if embracing a tree, with the palms facing inward about two feet from the chest. The participants look forward or slightly downward and chooses a high, medium, or low stance, depending on individual fitness. The feet are planted firmly on the ground as if standing on a well stone; deep rooted and unshakable. Breath adjustment starts with a normal natural breath and gradually transitions to abdominal breathing and reverse abdominal breathing, with a slower respiratory rhythm and rate and increased volume and depth as the posture gradually lowers. Mind adjustment requires concentrating on the three circular configurations with a relaxed demeanor.

## Sham-controlled Post Standing group I (TCPS posture with natural chest breath adjustment)

Sham-controlled *Post Standing* group I also consists of three elements: body, breath, and mind adjustment, where the body and mind adjustment training are the same as the standardized TCPS exercise. However, breath adjustment requires mainly using natural chest breathing and then only regulating breathing to make it slow, fine, and continuous.

## Sham-controlled Post Standing group II (normal standing posture with abdominal breathing adjustment)

Sham-controlled *Post Standing* group II training again contains the three elements of body, breath, and mind adjustment. Here, body adjustment requires standing erect in a natural manner with a step sideways with the left foot such that the two feet are shoulder-width (or a little wider) and parallel with each other. The knees will be bent slightly, the hips relaxed, and the abdomen contracted. Arms will be hung loosely at the subject’s sides, with the palms facing inwards and elbows slightly bent. The fingers will be spread apart and the fingers slightly bent at the joints so that the palms are cupped and about 15 cm from the body. Breathing training is the same as standardized *TCPS*, and mind adjustment requires concentrating on the normal standing posture with a relaxed demeanor.

## Sham-controlled Post Standing group III (normal standing posture with natural chest breath adjustment)

Sham-controlled *Post Standing* group III training has the same requirements as sham-*Post Standing* group II training, except for breathing adjustment training, which will use natural chest breathing and then only regulating breathing to make it slow, fine, and continuous.

### Training quality monitoring and duration

The 14-week training program will include both centralized and supervised training. Centralized training will last two weeks, with five 30-min sessions per week. Participants in the different groups will learn the key adjustments known as “regulating body, breath, and mind” and all ancillary practices until they master them under the guidance of coaches. After centralized training, participants will practice supervised training for the remaining 12 weeks, with five 30-min sessions per week. Coaches will supervise training to ensure correct practice. Participants will be required to complete a training self-evaluation log, which will include assessing the difficulty of training; the level of relaxation or tension or pain and fatigue in different parts of the body; perspiration levels; saliva secretion; body warmth sensation; breath control state; meditative state of mind; and any abnormal physical and mental reactions during and after training.

Feasibility will be assessed based on the number and content of training self-evaluation log entries per day. Compliance and attendance will also be monitored using training sign-in sheets to assess attendance rates. To eliminate bias from excessive physical activity (PA), all participants in both groups will be asked to record their PA (type and intensity of activities, duration of sedentary time, and sleeping hours) in diaries during the training period.

### Participant recruitment and enrolment

Participants will be recruited using various methods including online social media platforms, schoolyard advertisements, recruitment posters, health education lectures, and school radio announcements. Additionally, leaflets and brochures will be distributed, providing a brief introduction to the trial and the researchers’ contact information. During the screening phase, interested college students will contact the research assistants by telephone or email. Two trained and accredited investigators will repeatedly assess their sleep quality using two assessment scales (PSQI and ISI) and then determine eligibility for participation. Research assistants will provide written information about the study and explain the objectives, specific procedures, potential adverse effects, and expected benefits to potential participants. They will also enquire about their interest in joining the study. If the applicant meets the study criteria, they will be required to sign a written informed consent form prior to enrolment. Additionally, participants will be informed that they can withdraw from the study at any time during the trial period. Each participant will receive a logbook to monitor their involvement and training practices, and they will be asked to report any adverse events (AEs).

### Blinding method

In this trial, the coaches delivering the training will not be involved in the outcome assessments but they cannot be blinded to group allocation. However, to blind the recruited participants, sham-controlled *Post Standing* groups will be established, with participants unaware of the features of Qigong exercises and basic mind–body standards and practices of TCPS. Prior to group allocation, all training coaches will discuss with each participant that all forms of training may benefit them. Four blinding codes, "A," "B," "C," or "D," will be used to blind the data collectors, outcome assessors, and statistician, who will not be involved in group allocation and training implementation.

### Randomization and allocation concealment

Participants will be randomized after baseline assessment. An independent statistician will use SPSS v23.0 (IBM Statistics, Armonk, NY) to generate the random allocation sequence, and the randomized group assignments will be stored securely in sealed envelopes by an independent researcher not involved in recruitment, evaluation, or training. After participants have provided informed consent and completed the evaluations, the envelopes will be opened and participants will be assigned to either the standardized TCPS group or one of the sham-controlled *Post Standing* groups.

#### Follow-up period

During the unsupervised follow-up period after the intervention, participants will resume their original lifestyles but will be asked to record their daily PA and stressful life events. Participants will submit these records to the researchers by email daily as follow-up. Sleep quality and quality of life will be re-evaluated using self-reported scales at the end of the four-week and eight-week follow-up periods.

## Outcome assessment

### General demographic characteristics

Baseline data collection will be collected one week prior to randomized allocation and will include general demographic characteristics including sex, age, nationality, height, weight, current disease, allergy history, medical history, family history, and medication use. Daily lifestyle factors including diet, physical activity habits, learning and living schedules, smoking and alcohol use, and participation in sports clubs and societies, as well as stressful life events, will also be recorded.

#### Efficacy outcome evaluation

Sleep quality will be measured using the PSQI and the ISI. The PSQI consists of 19 questions about sleep habits over a month measuring seven components including sleep quality, duration, and disturbances. Scores range from 0 to 21, with a cutoff of 5 indicating insomnia or clinical sleep impairment [28, 29]. The ISI is a self-reported questionnaire measuring the severity of insomnia and functional impairments in the previous month. The total score on the ISI can range from 0 to 28 points. A score of 0 to 7 indicates no clinically significant insomnia, 8 to 14 is regarded as mild insomnia, 15 to 21 is regarded as moderate insomnia, and ≥ 22 points is regarded as severe clinical insomnia [30, 31].

Quality of life will be assessed using the 36-Item Short Form Health Survey Questionnaire (SF-36), a validated 36-item questionnaire containing sections on physical functioning, role-physical, body pain, general health status, vitality, social functioning, role-emotional, mental health, and health transition. Total scores on each scale are transformed and can range from 0 to 100, where a higher score indicates better self-perceived health status [32, 33].

#### Neurophysiological assessment

A resting EEG is a graphical representation of the electrical activity of a group of brain cells. It is obtained by recording and amplifying the brain's spontaneous biopotentials from the scalp using advanced electronic equipment [24–27]. This study will use the Nuamps 40-channel EEG system and Curry 7 acquisition software (Compumedics, Charlotte, NC, United States). The sampling frequency will be set at 1000 Hz and the band-pass filter ranges from 0.01 to 100 Hz. EEG data will be preprocessed using the EEG MATLAB toolkit in MATLAB (R2012a, MathWorks, Natick, MA, United States). Power spectrum analysis will be used to measure the EEG, which quantifies the activity in different frequency bands of delta (2–4 Hz), theta (4–8 Hz), alpha1 (8–10.5 Hz), alpha2 (10.5–13 Hz), beta1 (13–20 Hz), beta2 (20–30 Hz), and gamma (30–40 Hz). The relative and absolute power levels of these frequency bands will be evaluated to assess brain activity and the basic neurophysiology of mind adjustment during TCPS. The study will also explore correlations within and between the hemispheres as well as the functional coupling between different brain areas to investigate the mechanisms underlying the effects of TCPS on insomnia.

#### Plantar pressure and biomechanical equilibrium and physical function assessments

The Footscan Balance 7.7 acquisition and recording system and Free STEP v.1.5.01 analysis software (Sensor Medica, Rome, Italy) will be used to evaluate plantar pressure and biomechanical equilibrium. A pressure sensing plate measuring 240 × 50 cm and a sampling frequency of 50 Hz will be used. The analysis will include average foot plantar pressure, peak foot plantar pressure, foot contact area, center foot plantar pressure, and biomechanical balance function index (center-of-gravity path, body swing amplitude, envelope area of posturography, and average barycentric coordinates). These measures will help to clarify the basic pathophysiology of body adjustment during TCPS [34, 35].

To measure physical function, the MP150 multiple-channel electrophysiological recording device (Upwards Teksystems Ltd., Hong Kong, China) will be utilized with a maximum sampling frequency of 400,000 points/second. The physical function measures of interest in this study include respiratory function, HRV, blood pressure, and blood oxygen saturation. Respiratory function measurements, including chest and abdominal amplitudes and movements and respiratory flow rate, will be obtained to clarify the pathophysiology of breath adjustment during TCPS [36, 37]. HRV, a sensitive and non-invasive indicator of autonomic nervous activity, will be analyzed using time-domain and frequency-domain parameters [38, 39] in combination with blood pressure [40, 41] and oxygen saturation [42, 43] measures to identify the impact of body and breath adjustments in TCPS on physical function.

#### Synchronous detection procedure

Different groups of participants will be required to follow the pre-recorded guided voice instructions to practice body, breath, and mind adjustments in sequence. It should be noted that all participants at baseline will simply imitate the training practice under the pre-recorded guided voice instructions. Synchronous detection, including plantar pressure and biomechanical balance, physical function, and neurophysiological assessments, will be performed during the three-minute resting and relaxed standing state before the training practice state with self-active adjustment, the five-minute standing practice state with self-active adjustment, and the three-minute resting and relaxed standing state after the standing practice state with self-active adjustment. The stability of the instrument during the synchronous detection procedure will also be recorded.

#### Reporting adverse events and safety evaluations

No adverse events have been reported during our preliminary investigations. However, if any adverse events occur during the study (such as headaches, dizziness, chest tightness, palpitations, muscle soreness, or other physical discomfort), whether related to the study or not, they will be recorded in detail, including the timing, severity, and management. If any serious adverse events occur, the researchers will immediately report them to the medical ethics committee, who will decide if the participant needs to withdraw from the study. The relationship between serious adverse events and intervention training will also be analyzed.

#### Data management and monitoring

Two professional research assistants will conduct quality control of data collection and be responsible for entering their files into the same database, crosschecking them for data accuracy, and storing it with password protection.

#### Statistical analysis

We will use IBM SPSS Statistics 25.0 (IBM Statistics, Armonk, NY) for statistical analysis, with a significance threshold of 0.05 and 95% confidence intervals for all estimates. Categorical variables will be reported as frequencies or percentages, while continuous variables will be described using mean ± standard deviation for normally distributed data and median [interquartile range (IQR)] for non-normally distributed data. We will conduct intention-to-treat (ITT) and per-protocol (PP) analyses for all outcomes. If ITT and PP results are consistent, we will consider them reliable; otherwise, ITT results will be preferred. For missing data, we will use multiple imputation and last observation carried forward analysis. Baseline demographic characteristics will be compared between groups using analysis of variance (ANOVA), non-parametric Wilcoxon signed rank, or chi-squared tests. Adjusted analysis with multiple linear regression or logistic regression will be performed if potential confounding factors are present. Repeated-measures ANOVA (group × time) will be conducted to compare outcome measures between the standardized TCPS and sham-controlled *Post Standing* groups and within the standardized TCPS group at different assessment time points. Safety will be assessed by recording and reporting adverse events for each group. A chi-squared or Fisher's exact test will be used to compare frequency differences in adverse events between groups, and severe adverse events will be described in detail. Logistic regression and linear mixed-effects models will be employed to explore factors affecting adherence and feasibility, adjusted for confounders. Adherence-related data will be obtained from training self-evaluation logs and sign-in sheets.

## Discussion

This protocol describes the first double-blind parallel RCT aiming to evaluate the efficacy, safety, and feasibility of using TCPS for sleep quality improvement in college students with insomnia. Compared with the diverse Qigong modalities used to treat insomnia in the literature, using TCPS to treat insomnia in college students has the following advantages. First, TCPS is a static form of Qigong that requires minimal space and can be practiced by college students in places like dormitories or outdoors. It can also be easily incorporated into the study day, such as during the commute or study breaks, thereby encouraging its practical application. Second, TCPS is easy to learn and has a low risk of injury, contingent upon adherence to the correct taught posture and respiratory principles. This makes it optimal for independent and persistent practice by college students. Third, within the university milieu, insomnia tends to emerge as a consequence of the psychophysiological consequences of academic obligations and sociocultural interactions. Our previous research has shown that TCPS has therapeutic effects on anxiety symptoms in the college population through regulation of respiratory function, correction of body balance, and augmentation of autonomic nervous system function [20–22]. Thus, TCPS has distinct advantages in ameliorating sleep disturbances amongst this specific demographic by bolstering both physical resilience and psychological welfare.

Regarding the clinical trial design, we attempt to enhance the validity and reliability of this trial by implementing the following key measures. First, this study will evaluate sleep quality and quality of life of college students at multiple time points including before, during, and after TCPS intervention training and follow-up through the sleep quality self-evaluation and quality of life scales, aiming to quantify the short- and long-term benefits of TCPS for insomnia. Second, we will establish three sham-controlled groups practicing fabricated TCPS regimens that separate the body and breath adjustment requirements to illustrate the advantages of synergizing the TCPS posture and abdominal breathing to modulate cerebral function and ameliorate insomnia. This will help to enhance lay comprehension of Qigong's core principle – achieving a state of harmony through the "three adjustments" of body, breath, and mind. Furthermore, we will use electrophysiological techniques to synchronously measure participants' biomechanical balance and plantar pressures, monitor their breathing and other physical parameters, and analyze their brain activity when engaging in TCPS, with the aim of evaluating consistency of participants' posture and breathing and whether they achieve the "three adjustments" of body, breath, and mind. Third, this study will reveal possible reasons why TCPS improves insomnia symptoms in college students, thereby obtaining more objective evidence to promote the application of TCPS in the management of insomnia.

Studies have shown that practicing Qigong exercises increases the alpha and theta frequency band in the frontal and the parietal cortices, suggesting that Qigong stimulates and regulates brain regions related to emotional management and sensory processing through physical movements [24, 25, 27], providing a potential neurobiological basis for the beneficial effects of Qigong in addressing psychological issues. Other than frequency, functional connectivity has been shown to match the mind adjustment of Qigong exercises [27, 42]. If TCPS has a positive effect on sleep quality, this may be reflected by an increase in functional connectivity within and between hemispheres following TCPS. Conversely, prolonged periods of stress are thought to lead to the sustained and abnormal activation of the hypothalamic–pituitary–adrenal axis, which exacerbates autonomic nervous system imbalance and ultimately culminates in chronic insomnia [45–47]. In that context, examining and evaluating respiratory function, HRV, blood pressure, and oxygen saturation could provide new information on autonomic nervous system balance and physiological reactions to help explain the physiological mechanism underlying the impact of TCPS on physical function.

However, the trial also has several potential limitations. The recruitment criteria are limited to college students aged 18–26 years with insomnia. Thus, the findings of study may not be generalizable to other age groups. Additionally, the optimal intensity and timing of TCPS remain unknown, but we believe that the TCPS scheme used in this trial will provide information for any further optimization studies. Despite these limitations, this trial represents an important milestone in the development of new therapeutic approaches for insomnia and will be easily implementable for college students with insomnia.

## Data Availability

No datasets were generated or analysed during the current study.

## Abbreviations

AE: Adverse event
ANOVA: Analysis of variance
CBT-I: Cognitive behavioral therapy for insomnia
CNS: Central nervous system
DSM-V: Diagnostic and Statistical Manual of Mental Disorders, Fifth Edition
EEG: Electroencephalography
HRV: Heart rate variability
IQR: Interquartile range
ISI: Insomnia Severity Index
ITT: Intention-to-treat
PA: Physical activity
PP: Per-protocol
PSQI: Pittsburgh Sleep Quality Index
RCT: Randomized controlled trial
SF-36: 36-Item Short Form Health Survey Questionnaire
SPIRIT: Standard Protocol Items: Recommendations for Interventional Trials
TCM: Traditional Chinese medicine
TCPS: Three-circle Post Standing
